# Supplementary figures and images for: Sex-specific and concentration-dependent influence of Cremophor RH 40 on ampicillin absorption via its effect on intestinal membrane transporters in rats
Source: PLoS One. 2022 Feb 28;17(2):e0263692. doi: 10.1371/journal.pone.0263692 (PMC8884507; doi:10.1371/journal.pone.0263692)

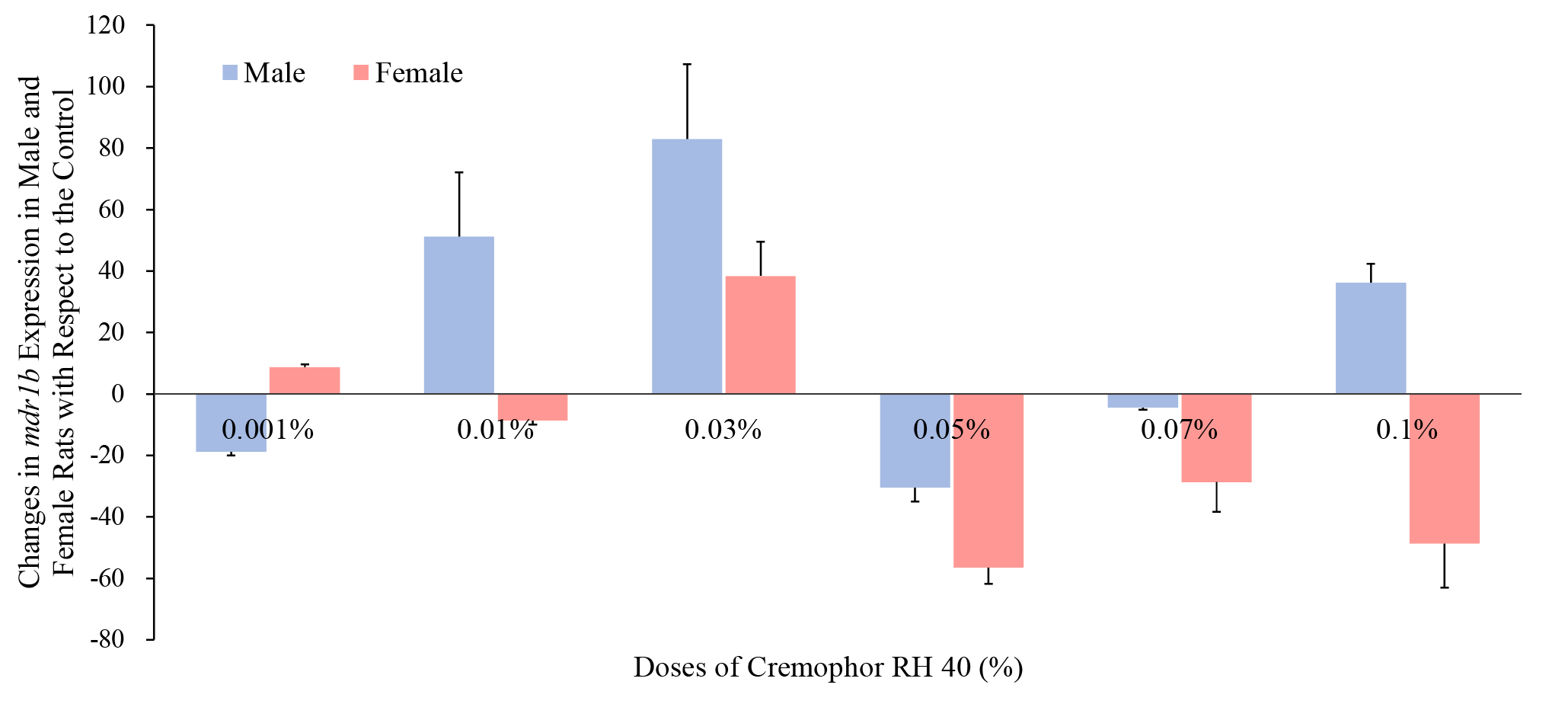

Supplement: S1 Fig — (TIF) [file pone.0263692.s001.tif]

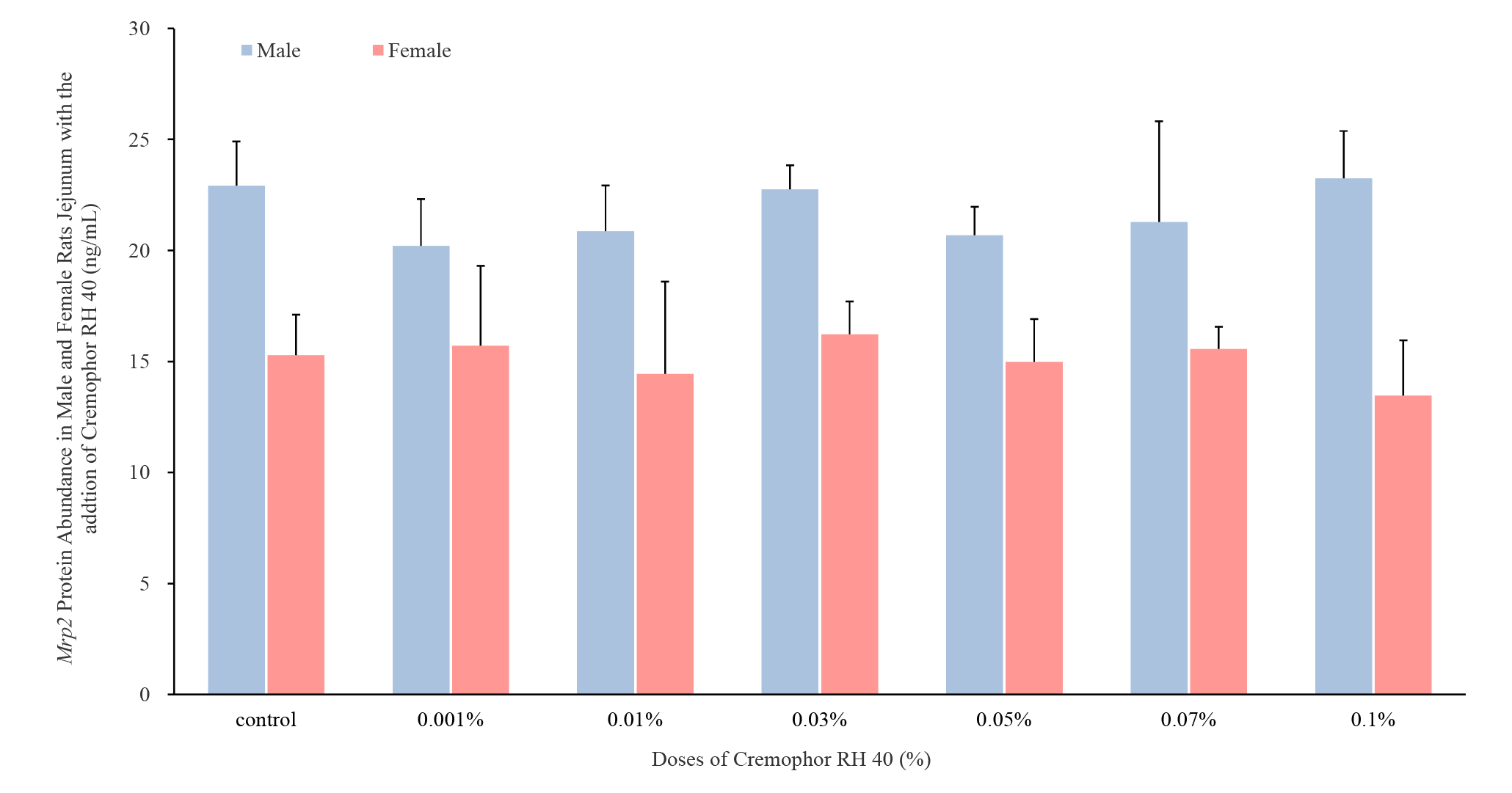

Supplement: S2 Fig — (TIF) [file pone.0263692.s002.tif]

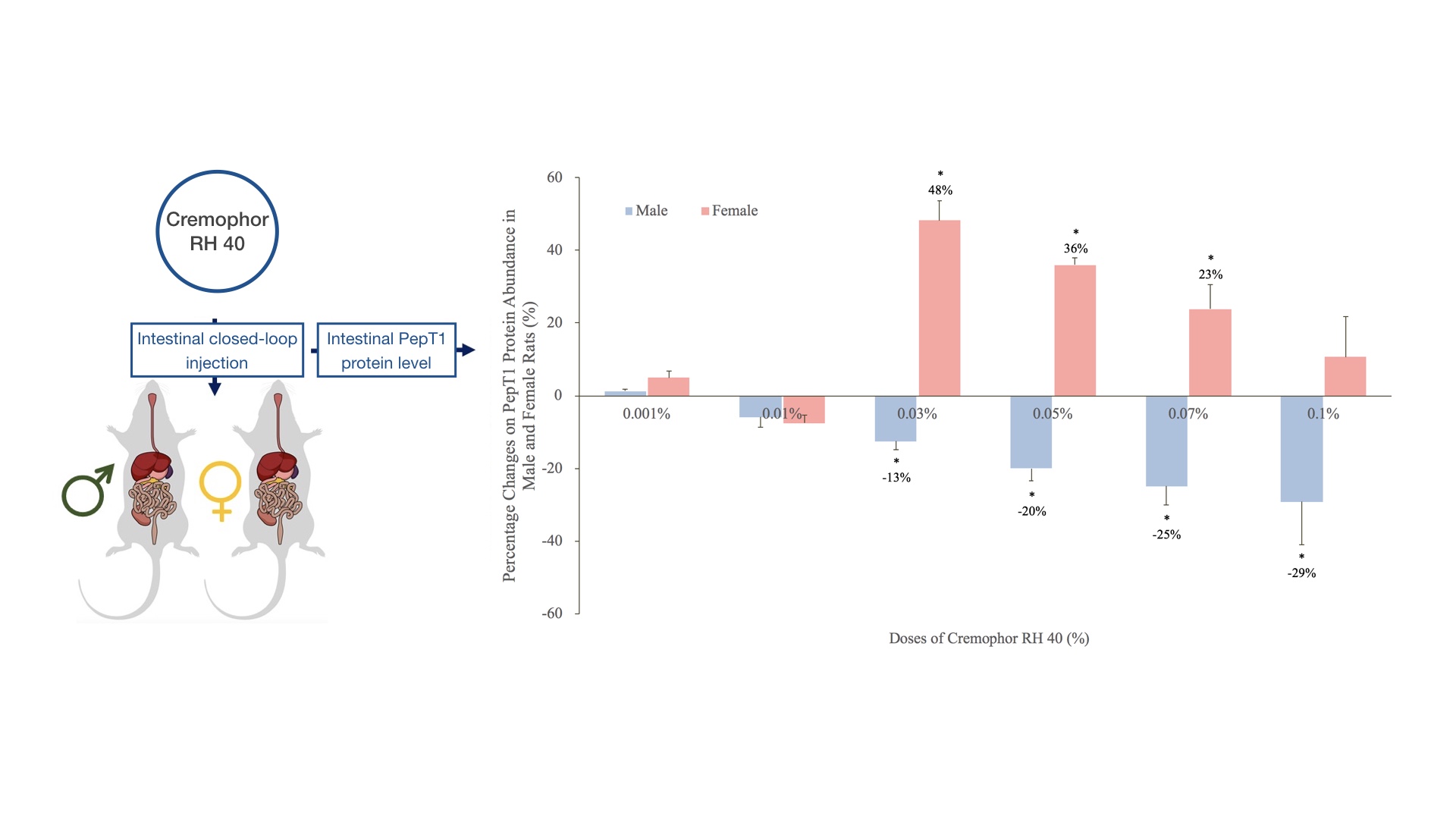

Supplement: S1 Graphical abstract — (JPEG) [file pone.0263692.s004.jpeg]
