# Supplementary material for: Sex-specific and concentration-dependent influence of Cremophor RH 40 on ampicillin absorption via its effect on intestinal membrane transporters in rats
Source: PLoS One. 2022 Feb 28;17(2):e0263692. doi: 10.1371/journal.pone.0263692 (PMC8884507; doi:10.1371/journal.pone.0263692)
Supplement: S1 Table — (DOCX) [file pone.0263692.s003.docx]

**Supporting Information**

**S1 Table** Effect of Cremophor RH 40 on the pharmacokinetic parameters of ampicillin in male and female Wistar rats (mean±standard deviation, n=4)

| **Pharmacokinetic Parameters** | **Cremophor RH 40 (%, v/v)** | | | | | | |
| --- | --- | --- | --- | --- | --- | --- | --- |
|  | **0 (control)** | **0.001%** | **0.01%** | **0.03%** | **0.05%** | **0.07%** | **0.1%** |
| ***Male*** | | | | | | | |
| AUC_0-240_ (μg.min /mL) | 4693.11 ± 1652.03 | 4289.59 ± 499.55 | 4340.05 ± 451.76 | 3899.37 ± 738.00 * | 3150.57 ± 128.98 * | 3742.17 ± 226.84 * | 4459.51 ± 596.02 |
| AUC_∞_ (μg.min /mL) | 5807.39 ± 1730.08 | 5939.49 ± 557.37 | 5669.55 ± 379.27 | 4896.59 ± 719.83 * | 4269.24 ± 137.90 * | 4270 ± 306.65 * | 5679.41 ± 629.91 |
| c_max_ (μg/mL) | 33.71 ± 2.76 | 37.30 ± 8.42 | 28.37 ± 3.75 | 23.22 ± 9.70 * | 20.82 ± 3.98 * | 24.92 ± 7.33 * | 29.65 ± 4.04 |
| t_max_ (min) | 110 ± 20 | 120 ± 33 | 140 ± 23 | 100 ± 23 | 110 ± 20 | 130 ± 20 | 140 ± 23 |
|  |  |  |  |  |  |  |  |
| ***Female*** | | | | | | | |
| AUC_0-240_ (μg.min/mL) | 785.53 ± 188.51 | 817.45 ± 268.63 | 893.51 ± 285.28 | 1111.44 ± 137.03 * | 1039.50 ± 16.48 * | 983.77 ± 34.93 * | 753.06 ± 72.25 |
| AUC_∞_ (μg.min/mL) | 930.02 ± 113.36 | 1051.02 ± 227.04 | 1218.79 ± 213.97 | 1639.80 ± 147.38 * | 1398.43 ± 29.24 * | 1207.85 ± 63.49 * | 912.87 ± 100.95 |
| c_max_ (μg/mL) | 7.70 ± 0.64 | 8.28 ± 1.93 | 8.57 ± 1.71 | 10.11 ± 0.84 * | 9.85 ± 0.43 * | 9.27 ± 0.92 * | 8.76 ± 0.94 |
| t_max_ (min) | 80 ± 33 | 107 ± 23 | 100 ± 23 | 90 ± 20 | 90 ± 38 | 93 ± 20 | 107 ± 20 |

* Values are statistically different between the control and Cremophor RH 40 groups at p<0.05.
