# Supplementary material for: Sex-specific and concentration-dependent influence of Cremophor RH 40 on ampicillin absorption via its effect on intestinal membrane transporters in rats
Source: PLoS One. 2022 Feb 28;17(2):e0263692. doi: 10.1371/journal.pone.0263692 (PMC8884507; doi:10.1371/journal.pone.0263692)
Supplement: Attachment — Submitted filename: Comments.docx [file pone.0263692.s005.docx]

Sex-specific and concentration-dependent influence of Cremophor RH 40 on ampicillin absorption via its effect on intestinal membrane transporters

|  | Line number | Comment |
| --- | --- | --- |
| 1 | Line 71-73 | Cite a reference number for this statement. |
| 2 | 101 | Define what is PBS |
| 3 | 104-107 | Provide the number of male rats and the number of female rats used for the study separately. In the section 2.3. |
| 4 | 127 | Define what is LHD |
| 5 | 143 | According to the manuscript pharmacokinetic parameters of Cmax, tmax, AUC0-240 and AUC∞ have been calculated. However, in the results section all these parameters except AUC0-240 have to be deduced by the reader from the Figure 1b. It is required to provide the results for these parameters with the Figure 1b or separately. |
| 6 | 145 | Describe the positive and negative controls used in determining the effect of cremophor RH 40 on intestinal membrane damage. |
| 7 | 164 | Remove “a” before at |
| 8 | 181 | Provide the reference number for MacLean's study here |
| 9 | 241-242 | It is not clear the shown plasma concentration values are corresponding to what inlet concentration/s of ampicillin. Provide this information both in the paragraph here and in Figure 1b |
| 10 | 255-257 | Rather than given a range of 0.03% - 0.07%, provide the 3 Cremophor RH 40 concentrations resulting 41%, 32% and 25% increase in ampicillin absorption |
| 11 | 310-312 | If this hypothesis of possible influence of PepT1 is used to explain ampicillin absorption in male rats, how the same argument to be used to explain same thing in female. Need a discussion with respect to female rats on the same line. |
| 12 | 318-321 | Solubility measurements done is not mentioned in the methodology and in the results section. Therefore, bringing a new result in the discussion is not acceptable. Include solubility measurement method and results in the relevant sections. |
| 13 | Figures | Poor contrasting and clarity. Need improvements |
| 14 | Figure 1 | Write the units of the y-axis |
