# Supplementary material for: Sex-specific and concentration-dependent influence of Cremophor RH 40 on ampicillin absorption via its effect on intestinal membrane transporters in rats
Source: PLoS One. 2022 Feb 28;17(2):e0263692. doi: 10.1371/journal.pone.0263692 (PMC8884507; doi:10.1371/journal.pone.0263692)
Supplement: Attachment — Submitted filename: Response to Reviewers.docx [file pone.0263692.s006.docx]

**PLOS ONE Manuscript Review**

**Manuscript ID: PONE-D-21-36367**

**Title: Sex-specific and concentration-dependent influence of Cremophor RH 40 on ampicillin absorption in rats via its effect on intestinal membrane transporters.**

We thank the reviewer for his/her comprehensive comments to our manuscript and for highlighting areas needing clarification and improvement.

We have amended the manuscript in accordance with the comments of the referee and provide a point-by-point response to the minor issues raised in the following.

***Reviewer 1:***

|  | **Line number** | **Reviewer Comments** | **Authors’ Response** |
| --- | --- | --- | --- |
| 1 |  | The topic is so interesting and the research findings are well presented. Authors had discussed their results appropriately and had selected good references to support it.  The only remark is that the resolution of Figure 1 needs improvement because it is not readable. | Thank you for thoroughly reading our manuscript and for highlighting areas needing clarification and improvement.  We have improved and updated Figure 1. |

***Reviewer 2:***

|  | **Line number** | **Reviewer Comments** | **Authors’ Response** |
| --- | --- | --- | --- |
| 1 | Line 71-73 | Cite a reference number for this statement. | Thank you for raising this concern. The reference has been added in line 80. |
| 2 | 101 | Define what is PBS | We thank the reviewer for this suggestion. The full title for PBS has been added. |
| 3 | 104-107 | Provide the number of male rats and the number of female rats used for the study separately. In the section 2.3. | We thank the reviewer for this comment. The number of rats used in this study has been updated in line 125-126. |
| 4 | 127 | Define what is LHD | We thank the reviewer for this suggestion. The full title for LHD has been added. |
| 5 | 143 | According to the manuscript pharmacokinetic parameters of C_max_, t_max_, AUC_0-240_ and AUC_∞_ have been calculated. However, in the results section all these parameters except AUC_0-240_ have to be deduced by the reader from the Figure 1b. It is required to provide the results for these parameters with the Figure 1b or separately. | Thank you for raising this concern. We have showed these data in an additional S1 Table. |
| 6 | 145 | Describe the positive and negative controls used in determining the effect of cremophor RH 40 on intestinal membrane damage. | Thank you for raising this concern.  We have enriched the methodology (line 163-164), and provided Supplementary Figures to support that there are no membrane damages led by Cremophor RH 40. |
| 7 | 164 | Remove “a” before at | Thank you very much for your suggestion. Language has been corrected. |
| 8 | 181 | Provide the reference number for MacLean's study here | Thank you for raising this concern. The reference has been added. |
| 9 | 241-242 | It is not clear the shown plasma concentration values are corresponding to what inlet concentration/s of ampicillin. Provide this information both in the paragraph here and in Figure 1b | We thank the reviewer for this suggestion. We have enriched the result paragraph (line 266) and captions of figures according to this comment (line 280). |
| 10 | 255-257 | Rather than given a range of 0.03% - 0.07%, provide the 3 Cremophor RH 40 concentrations resulting 41%, 32% and 25% increase in ampicillin absorption | We thank the reviewer for this suggestion. We have amended the Figures in main text and put the results on other concentrations in the Supplementary information. |
| 11 | 310-312 | If this hypothesis of possible influence of PepT1 is used to explain ampicillin absorption in male rats, how the same argument to be used to explain same thing in female. Need a discussion with respect to female rats on the same line. | We thank the reviewer for this comment.  The results observed herein are about comparation on two transporters, P-gp and PepT1, between male and female rats. Thus, we have enriched our discussion on female rats in the same paragraph (line 382-387). |
| 12 | 318-321 | Solubility measurements done is not mentioned in the methodology and in the results section. Therefore, bringing a new result in the discussion is not acceptable. Include solubility measurement method and results in the relevant sections. | We thank the reviewer for this comment. The methodology has been enriched regarding the solubility measurements (line 116-123). |
| 13 | Figures | Poor contrasting and clarity. Need improvements | We thank the reviewer for this suggestion. All the figures have been re-edited and provided in a better quality. |
| 14 | Figure 1 | Write the units of the y-axis | Thank you for raising this concern. The units of the y-axis have been added. |

***Reviewer 3:***

|  | **Line number** | **Reviewer Comments** | **Authors’ Response** |
| --- | --- | --- | --- |
| 1 |  | It seems irrational or inexactly that the dose of Cremophor RH 40 in the experiment was noted as volume in volume. Also the weight dose of ingredients administrated orally was preferred rather than volume. | We thank the reviewer for raising this concern. We apologize that here is a typo, the unit is w/v, which has corrected in the manuscript (line 115 and line 353). |
| 2 | Line 299 | Explanations with more details should be available. | Thank you for this suggestion. We have enriched the explanations (line 368-372). |
| 3 |  | The pharmacokinetic parameters (C_max_, t_max_, AUC_0-240_ and AUC_∞_) should be listed as a table form in the manuscript. | We thank the reviewer for raising this comment. We have showed these data in an S1 Table. |
| 4 | Line 92,  164, 267, etc. | Some spelling mistakes should be well corrected. | Thank you for raising this concern. These spelling mistakes have been corrected. |

***Reviewer 4:***

|  | **Line number** | **Reviewer Comments** | **Authors’ Response** |
| --- | --- | --- | --- |
| 1 |  | Addition the of the word (in rats) at the end of the title. | We thank the reviewer for this suggestion. The title has modified as “*Sex-specific and concentration-dependent influence of Cremophor RH 40 on ampicillin absorption via its effect on intestinal membrane transporters in rats* ”. |
| 2 |  | Add information about the excipient Cremophor RH 40 and its physicochemical properties in the introduction part | Thank you for this suggestion. The usage and physicochemical properties of Cremophor RH 40 was added in the introduction part in line 80-84. |
| 3 |  | Why Cremophor RH 40 has been choose and not Cremophor EL as an excipient? Did the second excipient also have the same effect on intestinal absorption of ampicillins? | We thank the reviewer for this question. Since Cremophor RH 40 has shown its sex-specific effect on efflux transporter P-glycoprotein, we chose Cremophor RH 40 instead of Cremophor EL in this study.  We agree that it is of vital important and interest to study more excipients, such as Cremophor EL, regarding their influences on drug bioavailability and intestinal transporters. Excipients with similar chemical structure or in one group could be studied in future work. |
| 4 |  | The selection of methods is rather broad and cover the design-space well as were the reported research results. | Thank you for raising this concern. We have modified the manuscript using Plos One template and optimized the structure. |
| 5 |  | Defined these abbreviations:  P6 - L 101: PBS P7 - L 127: LDH P11- L 208: HEPES P 12 - L 221: Tma-DPH P 16 -L 325: CMC | We thank the reviewer for this suggestion. The full title for PBS, LDH, HEPES, Tma-DPH, CMC have been added. |

***Reviewer 5:***

|  | **Line number** | **Reviewer Comments** | **Authors’ Response** |
| --- | --- | --- | --- |
| 1 |  | Please explain the basis of the Cremophor RH 40 dosage used in the article and its relationship with the clinical preparations | We thank the reviewer for this question. The studied concentrations of Cremophor RH 40 were chosen from *Handbook of Pharmaceutical Excipients (Six edition)*, which were used in the commercial and/or lab formulation developments. |
| 2 | Line 299 | Transport experiments on Caco-2 cells are needed to verify the effects of Cremophor RH 40 on the PepT1 protein recruitment on the cell membrane | We thank the reviewer for this question. We did want to confirm the mechanisms on the sex-specific effects of Cremophor RH 40 on intestinal transporters, the best methods and models were definitely Caco-2 cells. However, the cell lines could not present the ‘sex’ feature, which impede the mechanism investigation.  Our ideas were to set up a ‘sex-related’ intestinal cell line and /or intestine permeation model, such as personal gut-on-a-chips. Verifying and using these new models, further and deeper mechanism could be investigated and confirmed. |
| 3 | Line 366 | Relevant references are missing. | We thank the reviewer for this suggestion. All the references have been checked and the missing one has been added. |
| 4 |  | Try to calculate/estimate the meaning of the interaction of Cremophor RH 40 and ampicillin observed in your experiments. Does the magnitude of interaction and doses reflect the clinical observations in literature? Or is it x-fold lower | We thank the reviewer for this suggestion and question.  This study designed based on the previous findings on sex-related influence of excipients on P-gp, but not the clinical findings regarding sex differences on bioavailability. Actually, we did want to collect some clinical results which showed sex differences, and also provided the formulation ingredients. However, the route was limited and these clinical data were private for the industries.  In any case, we enriched the discussion part by estimating these interactions in clinical observation and the practical meaning of these interaction (line 483~490). |
